# Precipitation of β-Mn in the Form of Widmanstätten Side-Plates in the Ferrite Matrix of an Fe–28.6 Mn–10.9 Al Alloy Steel

**DOI:** 10.3390/ma19010133

**Published:** 2025-12-30

**Authors:** Rosemary Chemeli Korir, Wei-Chun Cheng

**Affiliations:** Department of Mechanical Engineering, National Taiwan University of Science and Technology, 43 Keelung Road, Section 4, Taipei 106, Taiwan

**Keywords:** Fe–Mn–Al alloy, phase evolution, ferrite phase, β-Mn precipitates, Widmanstätten morphology

## Abstract

**Highlights:**

- The Fe–28.6 Mn–10.9 Al alloy is BCC ferrite at temperatures between 850 and 1100 °C.
- β-Mn exists in the ferrite at temperatures ranging from 500 °C to 850 °C.
- β-Mn phase is formed as Widmanstätten side-plates.
- The formation of the β-Mn in BCC shows the following OR: ($02\bar{1}$)_β_ // (100)_α_ and [$\bar{1}12$]_β_ // [012]α.

**What are the main findings?**

- The alloy exhibits a single BCC phase at temperatures above 850 °C.
- β-Mn is thermally stable between 500 °C and 850 °C.
- β-Mn appears as Widmanstätten side-plates that coarsen with temperature.
- The β-Mn in BCC shows an OR established as ($02\bar{1}$)_β_ // (100)_α_ and [$\bar{1}12$]_β_ // [012]α.

**What are the implications of the main findings?**

- We clarify the precipitation behavior of β-Mn.
- We provide new insights into β-Mn phase stability in Fe–Mn–Al alloy.
- We provide information contributing to the development of high-strength, lightweight steels.

**Abstract:**

The microstructural evolution and phase stability in Fe–Mn–Al alloys play a decisive role in determining their mechanical performance and potential applications. This study investigates the precipitation behavior and crystallography of the β-Mn phase in an Fe–28.6 Mn–10.9 Al (wt.%) alloy subjected to annealing at 1100 °C, followed by water quenching and subsequent isothermal holding at temperatures between 500 °C and 900 °C for 20 h. Microstructural analysis using X-ray diffraction, optical and electron microscopy revealed a single body-centered cubic (BCC) ferritic matrix above 850 °C and the formation of β-Mn precipitates with Widmanstätten side-plate morphology at lower temperatures. The β-Mn phase was thermally stable between ~500 °C and 850 °C, with the volume fraction increasing with temperature and reaching a maximum near 650 °C. The β-Mn precipitates coarsened progressively with increasing temperature and were found to be richer in Mn than the surrounding Fe-rich BCC matrix. Crystallographic analysis established an orientation relationship (OR) of ($02\bar{1}$)_β_ // (100)_α_ and [$\bar{1}12$]_β_ // [012]α, where // denotes nearly parallel alignment, signifying a semi-coherent interface between the two structures. These findings clarify β-Mn precipitation, its interfacial relationship with ferrite, and its thermal stability in high-Mn Fe–Mn–Al alloys, offering guidance for microstructural design in next-generation lightweight steels.

## 1. Introduction

Fe–Mn–Al alloys have emerged as promising candidates for advanced high-strength, lightweight steels for automotive and structural applications [1,2,3]. The growing demand for improved fuel efficiency and reduced greenhouse gas emissions has intensified research into structural materials that combine low density with high strength and excellent formability [1,4]. Fe–Mn–Al alloys uniquely fulfill these requirements by offering up to 15–20% lower density than conventional steels while maintaining high tensile strength exceeding 800 MPa and superior corrosion resistance [2,5,6,7,8,9]. These attributes make them particularly suitable for automotive components where weight reduction directly translates into enhanced performance and energy savings [2,10]. Furthermore, the wide range of phase transformations and microstructural phenomena such as κ-carbide precipitation, austenite-to-ferrite transformations, and the formation of ordered B2 and D0_3_ phase provide exceptional tunability of mechanical and physical properties through compositional and heat treatment control [4,11,12]. Consequently, Fe–Mn–Al alloys are increasingly recognized as strategic materials that can bridge the gap between advanced high-strength steels and light alloys such as aluminum or titanium for sustainable automotive design [3,8,13].

The Fe–Mn–Al alloy system exhibits a rich variety of phase equilibria and transformation pathways, resulting in complex microstructures that strongly influence mechanical performance. Depending on the alloy composition and heat treatment conditions, a variety of stable and metastable phases can form, including disordered body-centered cubic (BCC) and face-centered cubic (FCC), ordered derivatives such as B2 (FeAl- or NiAl-type) and D0_3_ (Fe_3_Al-type), κ-carbide (Fe,Mn)_3_AlC), and, in certain high-Mn alloys, the β-Mn phase [4,5,9,11,12,13,14,15,16]. Other possible constituents include hexagonal close-packed (HCP, ε) and/or body-centered tetragonal (BCT, α’) martensite [17,18,19,20]. At high temperatures, a single-phase BCC or FCC matrix is generally stable, whereas cooling promotes decomposition through phase separation, ordering, or precipitation reactions, most notably the formation of κ-carbide within an FCC matrix or the development of B2/D0_3_ order within a BCC matrix [10,12,16]. These transformations are highly sensitive to the local Mn and Al concentrations, which control the stability fields of each phase and determine whether the microstructure remains ductile or becomes prone to brittleness [5,21,22]. Consequently, understanding the sequence and kinetics of phase evolution is essential for controlling microstructural stability and optimizing the balance between strength, ductility, and toughness in Fe–Mn–Al alloys.

Despite the promising lightweight and strengthening potential of Fe–Mn–Al systems, challenges persist in achieving thermally stable microstructures free from brittle phases that compromise mechanical integrity. Among these phases, the β-Mn phase has been widely identified as a critical concern, particularly in high-Mn Fe–Mn–Al compositions, where it frequently forms along grain boundaries or within inter-dendritic regions during high-temperature exposure or slow cooling [9,15,23,24,25,26,27,28]. This phase possesses a complex primitive cubic structure (space group P4_1_32) and is thermodynamically stabilized at intermediate temperatures in high-Mn compositions, often at the expense of the ductile BCC/FCC matrix [16,21]. Its precipitation not only depletes solute elements essential for solid-solution and precipitation strengthening but also introduces brittle interfaces that severely degrade toughness and ductility [16]. Therefore, understanding the thermal conditions that promote β-Mn formation and its interaction with other competing phases is important for establishing microstructural control strategies to suppress its occurrence. The present study investigates the precipitation behavior, crystallography, and phase stability of β-Mn in high-Mn ferritic Fe–Mn–Al alloys to advance our understanding and guide microstructural design in this alloy system.

## 2. Materials and Methods

An Fe–28.6 Mn–10.9 Al (wt.%) alloy ingot was prepared by vacuum induction melting, employing electrolytic iron and electrolytic manganese together with high-purity aluminum. We specify that all compositions in this work are reported in weight percent (wt.%). The melt was cast into ingots, homogenized at 1200 °C for 4 h, air-cooled, sectioned into billets, and hot-forged into slabs. Specimens measuring 15 mm × 10 mm × 2 mm were cut from these slabs for subsequent heat treatments and microstructural analyses. Prior to heat treatments, the specimens were mechanically ground to remove oxide layer and sealed under vacuum in quartz tubes to prevent oxidation and loss of the alloy’s constituent elements.

All the samples were annealed at 1100 °C for 1 h and then quenched in water at room temperature. The annealed samples were subsequently isothermally held at temperatures ranging from 500 °C to 900 °C for 20 h, followed by water quenching. In the following sections, the annealed sample will be referred to as the as-quenched sample. The heat-treated specimens were mechanically ground to prepare them for various microstructural analyses. For X-ray diffraction (XRD) analysis, the specimens were further ground into a fine powder to minimize texture effects and ensure random crystallographic orientation during measurement. The optical microscopy (OM) and scanning electron microscopy (SEM) analysis specimens were further polished using alumna suspensions, followed by etching in a 3% nital solution to reveal microstructural features. The overall microstructure was characterized by optical microscopy (OM) using an Olympus BX41M microscope (Olympus Corporation, Tokyo, Japan). Morphological and compositional analyses were performed using a JEOL JXA-7900SX high-resolution field-emission SEM equipped with an energy-dispersive X-ray spectroscope (EDS). Phases identification was carried out by XRD using a Bruker D2-PHASER diffractometer (second generation), operated at 30 kV and 10 mA, with Cu Kα radiation (λ = 1.5406 Å).

For higher-resolution microstructural and crystallographic analysis, a Talos F200XG2 transmission electron microscope (TEM) operated at 200 kV acceleration voltage was utilized. TEM-STEM imaging was performed in high-angle annular dark-field (HAADF) mode to provide both structural and compositional contrast, with HAADF enabling z-contrast imaging for atomic number-based differentiation of elements. The high-resolution elemental mapping and compositional analysis was conducted by EDX using a Super-X EDX detector integrated with the TEM system. The collected EDX data were processed and quantified using Thermo Scientific Velox software (version 6.0). TEM specimens were prepared by mechanically grinding the alloy samples to a thickness of approximately 80 μm, followed by punching out 3 mm diameter disks. These disks were then thinned to electron transparency using a twin-jet electro-polisher in a solution of 10% perchloric acid in 95% ethanol at −15 °C.

## 3. Results and Discussion

**Figure 1:**
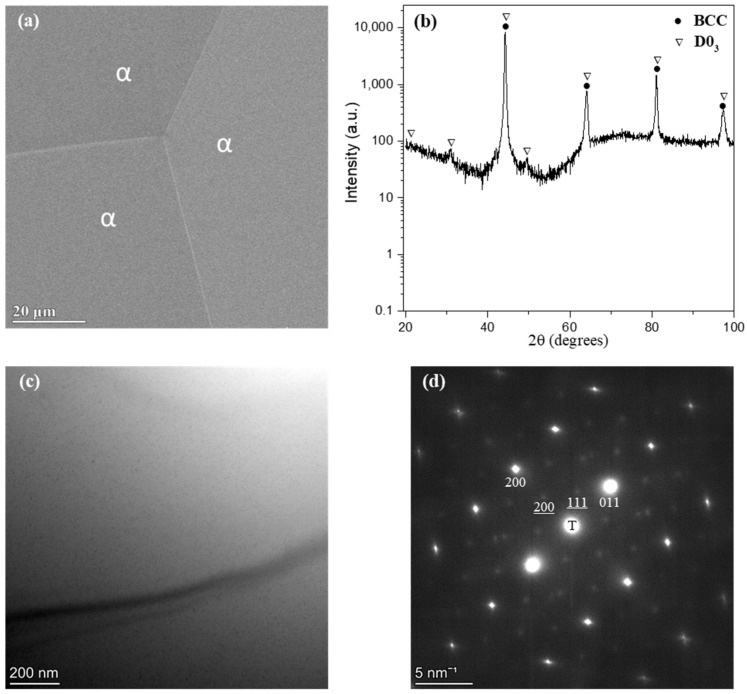
Microstructural characterization of the as-quenched Fe-28.6 Mn-10.9 Al alloy: (**a**) SEM image showing a homogeneous matrix marked as α; (**b**) XRD pattern (intensity plotted on a logarithmic scale) revealing BCC peaks with weak D0_3_ reflections; (**c**) TEM bright-field image confirming a uniform matrix; (**d**) SADP along BCC $\left[01\bar{1}\right]$ zone axis showing fundamental BCC and faint D0_3_ superlattice spots. The Miller indices corresponding to the D0_3_ phase are underlined. *T denotes the transmitted beam*.

The microstructural characterization of the as-quenched Fe–28.6 Mn–10.9 Al alloy is presented in Figure 1. The SEM secondary electron image (SEI) in Figure 1a reveals a homogeneous microstructure, indicating the absence of discernible second-phase precipitates. The corresponding XRD pattern in Figure 1b, plotted on a logarithmic intensity scale to enhance peak visibility, exhibits diffraction peaks characteristic of a BCC structure together with weak superlattice reflections attributed to the ordered D0_3_ phase. The calculated BCC lattice parameter of 0.2897 ± 0.0003 nm is consistent with values reported for ferritic Fe–Mn–Al alloys [29]. The TEM bright-field (BF) image in Figure 1c further confirms a uniform matrix, consistent with the SEM observations. The selected-area diffraction pattern (SADP) taken along the BCC $\left[01\bar{1}\right]$ zone axis in Figure 1d displays strong fundamental BCC reflections together with weak D0_3_ superlattice spots. The Miller indices corresponding to the D0_3_ diffraction spots are underlined to distinguish them from the BCC reflections. These observations indicate that the as-quenched is dominated by a ferritic BCC matrix with a minor degree of D0_3_-type atomic ordering.

On this basis, the results in Figure 1 confirm that the Fe–28.6 Mn–10.9 Al alloy retains a ferritic BCC structure after annealing at 1100 °C for 1 h and subsequent quenching. The dominance of the BCC phase is attributed to the high Al content, which stabilizes the ferritic structure in Fe–Mn–Al alloys [5,9]. The presence of weak D0_3_ superlattice reflections suggests partial atomic ordering within the BCC matrix during cooling, arising from the redistribution of Al and Mn atoms driven by their strong chemical affinity with Fe. driven by their strong chemical affinity with Fe. Similar ordering behavior has been reported in Fe–Mn–Al and Fe–Al alloys [27,30,31,32], where D0_3_ ordering cannot be completely suppressed by quenching. Thus, the formation of a fine D0_3_ phase during cooling contributes to microstructural stability while preserving the ferritic matrix.

**Figure 2:**
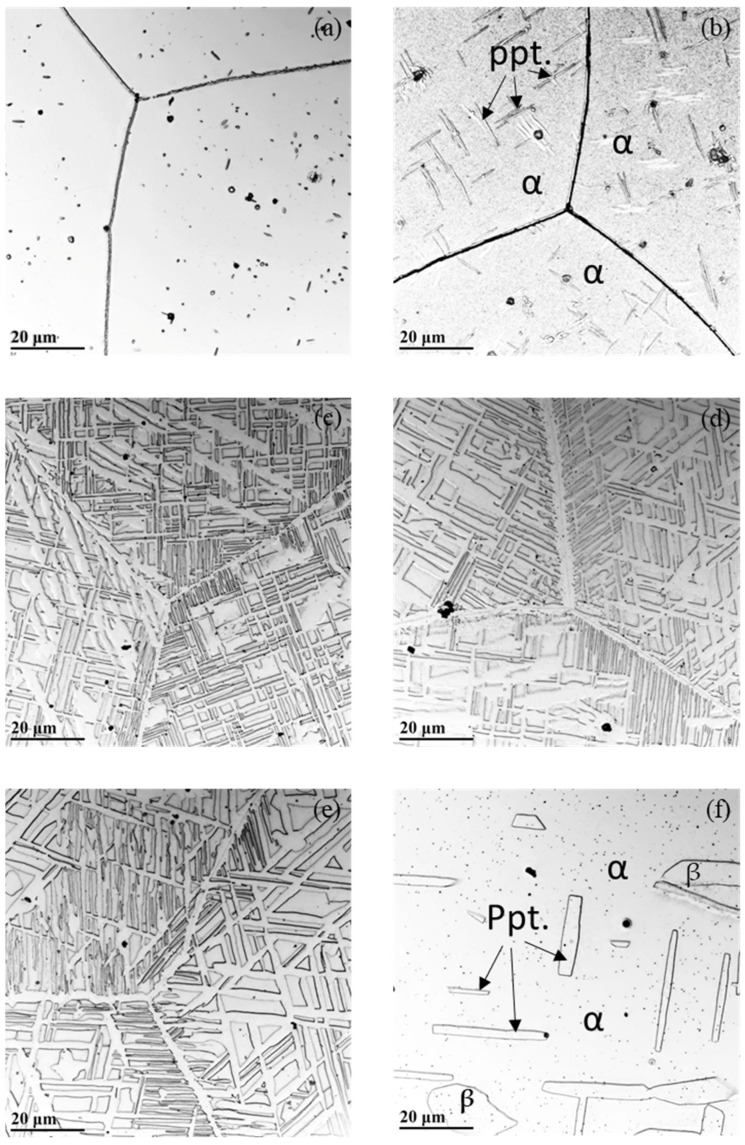
OMs of the as-quenched alloy after isothermal holding at various low temperatures as follows: (**a**) 500 °C, (**b**) 550 °C, (**c**) 600 °C, (**d**) 650 °C, (**e**) 700 °C, and (**f**) 800 °C. The images illustrate the appearance of second-phase precipitates labeled as β in the α matrix. The precipitate size increases progressively as the holding temperature rises, and the volume fraction of the precipitates is observed to reach its highest value at approximately 650 °C.

Figure 2 shows optical micrographs (OMs) of the alloy held isothermally at temperatures between 500 °C and 800 °C for 20 h. At 500 °C (Figure 2a), fine, sparsely distributed dot- and short needle-shaped second-phase precipitates are observed both within grains and along grain boundaries, indicating an early stage of precipitation. When the alloy was held at 550 °C (Figure 2b), precipitation becomes more pronounced, with the second phase developing into discrete clusters composed of lamellar layers. These clusters remain sparsely distributed within the grains but exhibit a clear directional alignment along specific crystallographic orientations. This second phase appear as fine plates, characteristic of a Widmanstätten-side-plate morphology, suggesting the establishment of a definite crystallographic orientation relationship with the surrounding matrix. Such directional alignment and plate-like geometry distinguish Widmanstätten precipitation from simple random precipitation, which typically occurs without a preferred crystallographic orientation or a well-defined interfacial relationship with the matrix.

Upon holding at 600 °C (Figure 2c) and 650 °C (Figure 2d), the second-phase precipitates formed large lamellar plates that extend across entire grains. The second-phase morphology evolves from micro-sized needles at temperatures below 550 °C to elongated plates at temperatures above 600 °C. Both the size and volume fraction of the precipitate increase as the temperature rises. The precipitate volume fraction reaches a maximum near 650 °C. When the holding temperatures is increased to 700 °C (Figure 2e), the precipitates appear coarsened. At 800 °C (Figure 2f), the precipitates become significantly larger but fewer than those at lower temperatures, reflecting enhanced coarsening behavior [33]. At 850 °C, no precipitates are observed, and at temperatures above this level the alloy reverts to a single-phase microstructure similar to the as-quenched condition shown in Figure 1a. The disappearance of second phase above 850 °C is consistent with thermodynamic predictions by Saha [21] and experimental observations reported by Bai et al. [9], indicating that second phase in duplex Fe–Mn–Al–C steel in the form of β-Mn is a metastable phase that dissolves upon approaching its solvus temperature. Consequently, the upper temperature limit for the stability of the second phase lies between approximately 825 °C and 850 °C and the alloy exists as a single ferritic BCC phase at temperatures above 850 °C. In the OM images, Figure 2b,f, the second-phase precipitates are labeled as β, and the surrounding BCC matrix as α, for clarity and consistent reference throughout the discussion.

**Figure 3:**
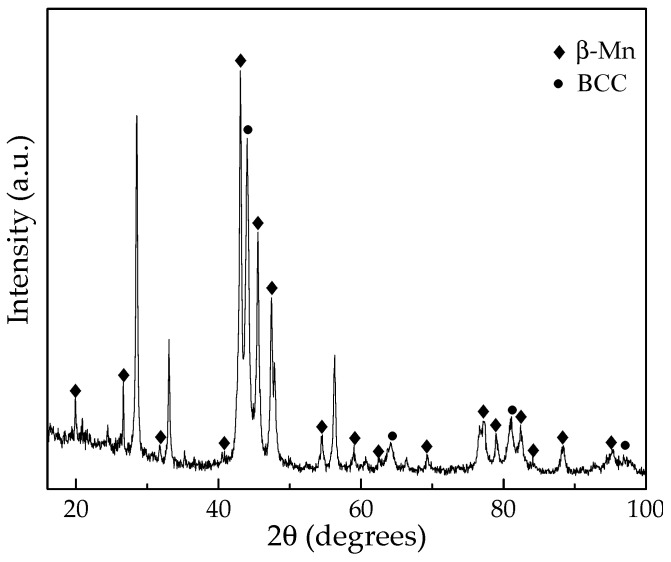
XRD pattern of the alloy after isothermal holding at 600 °C for 20 h. The pattern exhibits distinct peaks from both the BCC matrix and the β-Mn phase, confirming the precipitation of β-Mn within the BCC matrix.

XRD analysis, shown in Figure 3, was performed on the alloy after isothermal holding at 600 °C to identify the constituent phases. The 600 °C sample was selected because it contained comparable proportions of the matrix and precipitate phases, allowing the second phase to be distinctly detected. As shown in the diffraction pattern, both BCC and β-Mn phases coexist. Characteristic β-Mn peaks appear at approximately 2θ = 43.02°, 45.38°, and 47.73°, alongside BCC reflections at 44.03° and 64.13°. Peaks without assigned labels are attributed to oxide phases. The β-Mn peaks are relatively intense, indicating a high proportion of this phase at 600 °C. This finding is consistent with the OM in Figure 2c, which also shows an increased volume fraction of the precipitate phase compared with the matrix phase. The calculated lattice parameters are 0.6310 ± 0.0004 nm for β-Mn and 0.2903 ± 0.0001 nm for BCC, consistent with previously reported values for Fe–Mn–Al alloys [27,29]. Compared with the XRD pattern of the as-quenched alloy (Figure 1b), the appearance of β-Mn reflections in the 600 °C sample confirms that β-Mn precipitated from the supersaturated BCC matrix during isothermal holding. Taken together with the OM results in Figure 2, these findings conclusively identify the second phase as β-Mn. Accordingly, both XRD and OM analyses demonstrate that β-Mn precipitates form within the BCC matrix during isothermal holding at temperatures between approximately 500 and 850 °C. 

Overall, the results in Figure 2 and Figure 3 demonstrate that β-Mn precipitation in ferritic Fe–Mn–Al alloys is strongly temperature-dependent. Fine β-Mn plates form between 500 °C and 700 °C through a diffusion-controlled process, following a semi-coherent orientation relationship with the ferritic matrix. At higher temperatures, β-Mn progressively coarsened and finally dissolves back into the matrix, restoring a homogeneous BCC structure. These findings are consistent with prior studies on Fe–Mn–Al steels [9,21,26,27] but extend them by confirming that comparable precipitation behavior occurs within a purely ferritic composition, establishing the temperature range over which this β-Mn phase can be stabilized and controlled through heat treatment.

**Figure 4:**
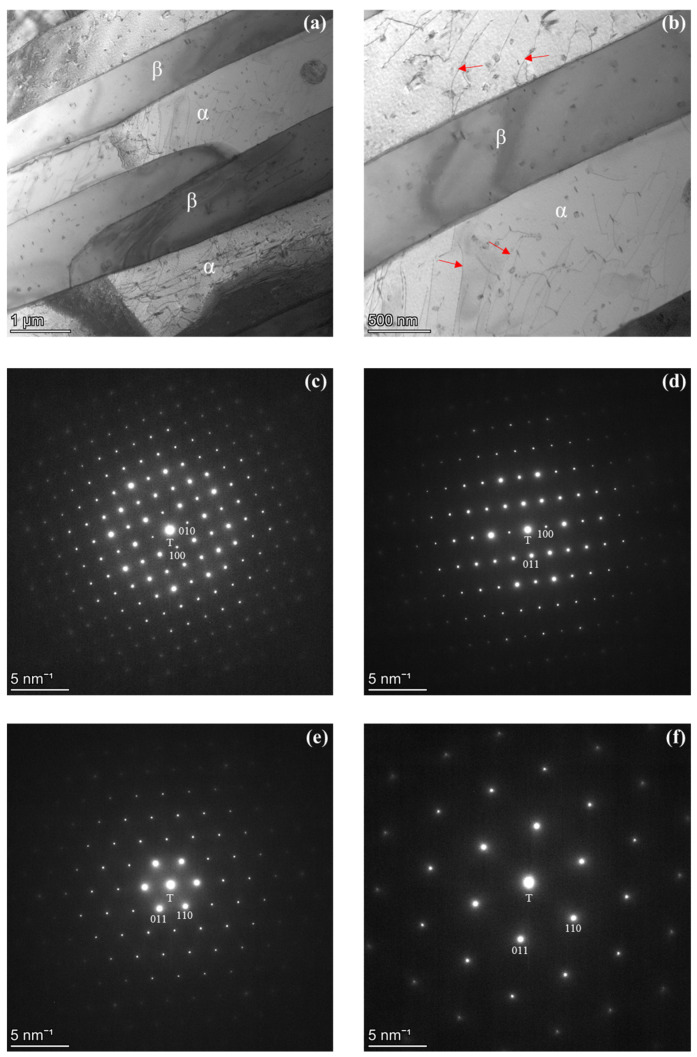
TEM characterization of the alloy after isothermal holding at 600 °C, confirming the formation of β-Mn precipitates within the BCC matrix. (**a**,**b**) BF images showing elongated β-Mn precipitates (β) embedded in the BCC matrix (α). Dislocations within the matrix, indicated by red arrows in (**b**), suggest the presence of coherency or semi-coherency strain. (**c**–**e**) SADPs acquired from the β-Mn phase along the [001], [0$\bar{1}$1], and [1$\bar{1}$1] zone axes, respectively. (**f**) SADP obtained from the BCC matrix along the [1$\bar{1}$1] zone axis. The combined imaging and electron diffraction results verify the coexistence of β-Mn and BCC phases.

TEM analyses were performed to verify the crystal structures and compositions of both the β-Mn and BCC phases. Figure 4 shows representative results from the alloy held at 600 °C. The BF images in Figure 4a,b reveal elongated, plate-like β-Mn precipitates embedded within the BCC matrix. These precipitates display a Widmanstätten-type morphology, aligning along specific crystallographic orientations of the matrix, which is typical of diffusion-controlled plate growth. Numerous dislocations are visible in the surrounding BCC matrix, as indicated by the red arrows in Figure 4b. The presence of the dislocations suggests that coherency or semi-coherency strains arose due to the lattice-parameter mismatch between β-Mn and the BCC matrix (as determined from the XRD results in Figure 3), resulting in local plastic relaxation rather than large-scale deformation of the matrix. To verify the crystal structure of the β-Mn phase, several SADPs were obtained from the precipitate in Figure 4b. The SADPs acquired along the [001], [0$\bar{1}$1], and [1$\bar{1}$1], zone axes of β-Mn (Figure 4c–e) exhibit the characteristic diffraction patterns corresponding to the β-Mn phase, confirming its complex primitive cubic structure. These observations are consistent with previous reports describing the β-Mn phase as a complex primitive cubic structure (space group *P4_1_32*) in Fe–Mn–Al alloys [9,27,28,34]. The SADP taken from the matrix along BCC along [1$\bar{1}$1] the BCC [1$\bar{1}$1] (Figure 4f) shows that the matrix retains a BCC structure. Collectively, these results provide direct crystallographic evidence that β-Mn precipitates nucleate and grow within the ferritic BCC matrix during isothermal holding at lower temperatures, leading to the coexistence of β-Mn and BCC phases in the alloy.

**Figure 5:**
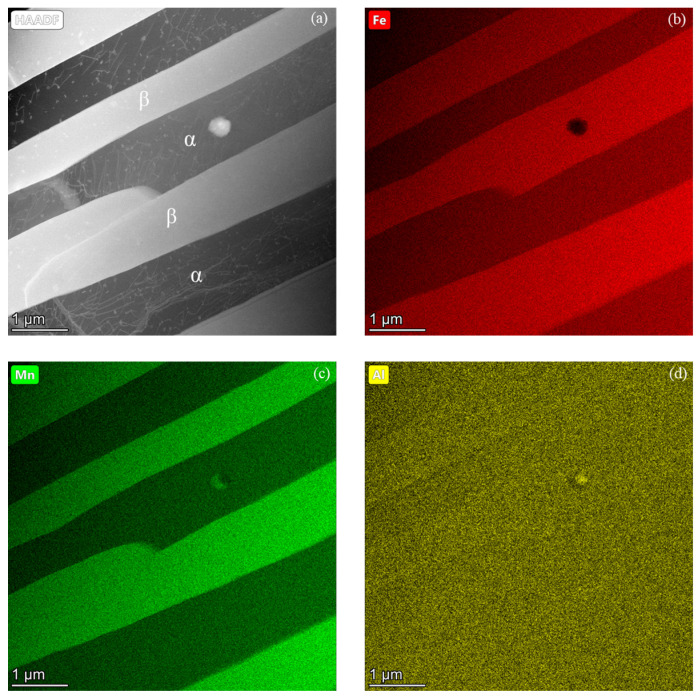
STEM-EDS analysis of the alloy subjected to the same heat treatment condition as in Figure 4, illustrating elemental partitioning between the BCC matrix and β-Mn precipitates during phase evolution: (**a**) HAADF image and (**b**–**d**) elemental mappings of Fe (red), Mn (green), and Al (yellow), respectively. The results reveal Fe enrichment in the BCC matrix and Mn enrichment in the β-Mn phase, while Al partitioning is relatively weak compared with the pronounced Fe and Mn partitioning.

After confirming the crystal structure of the β-Mn phase, further TEM analysis was performed to examine compositional partitioning between the precipitates and the matrix, as shown in Figure 5. The region analyzed in Figure 5 is located slightly above and to the right of the BF image area shown in Figure 4a. The elemental distribution mappings in Figure 5 provide evidence of compositional partitioning between β-Mn precipitates and the BCC matrix. The HAADF image in Figure 5a shows alternating regions of contrast corresponding to the dark BCC matrix and bright β-Mn precipitates. The BCC matrix phase is also confirmed by the presence of dislocations as was identified in Figure 4b. The color-coded EDS mappings in Figure 5b–d, corresponding to Fe (red), Mn (green), and Al (yellow), reveal pronounced chemical partitioning. Fe is enriched in the BCC matrix whereas Mn is strongly concentrated in the β-Mn precipitates, indicating preferential elemental partitioning during phase evolution. In contrast, Al exhibits a nearly uniform distribution, though it is slightly enriched in the β-Mn precipitates, suggesting limited partitioning under the current heat-treatment conditions. Quantitative EDS analysis yields average compositions of Fe–39.2 Mn–10.5 Al for the β-Mn phase and Fe–21.3 Mn–11.2 Al for the BCC matrix. These results indicate that the β-Mn phase is significantly enriched in Mn, whereas the BCC matrix is correspondingly enriched in Fe, corroborating the segregation behavior described by Chen et al. [26]. According to the binary Fe–Mn phase diagram, the minimum Mn content required to stabilize the β-Mn phase is approximately 60 wt.%, while the maximum Mn solubility in BCC ferrite is limited to ~4 wt.% [35]. The present compositional distribution analyses result therefore demonstrate that Al plays a critical role in extending the Mn solubility ranges of both the BCC and β-Mn phases, thereby stabilizing β-Mn at substantially lower Mn contents and enhancing Mn retention within the BCC matrix. These findings, together with the TEM observations in Figure 5, confirm that β-Mn formation during low-temperature isothermal holding is accompanied by pronounced elemental partitioning.

**Figure 6:**
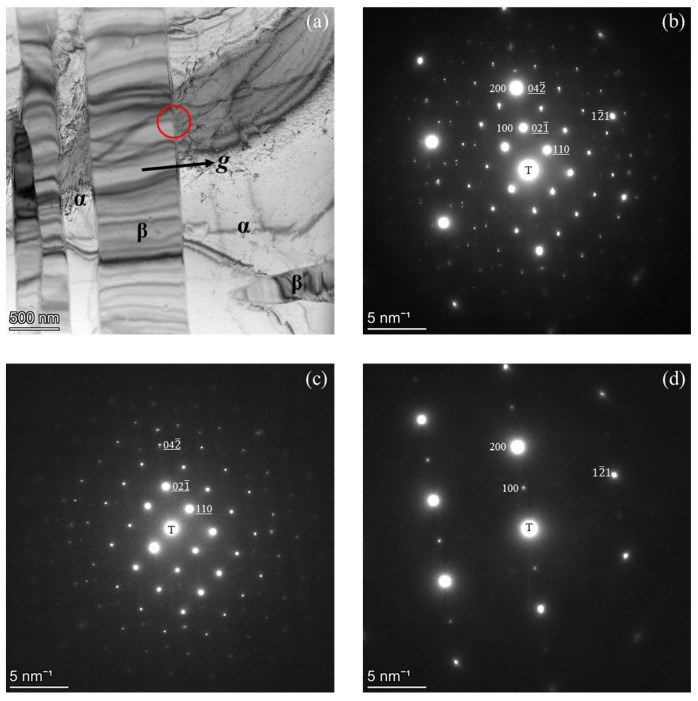
TEM analysis showing the ORs between the β-Mn and BCC phases in the alloy held isothermally at 550 °C: (**a**) BF image revealing β-Mn precipitates within the BCC matrix; (**b**) SADP obtained from the red-circled region in (**a**) containing both phases; (**c**) SADP from the β-Mn region along [$\bar{1}12$]; and (**d**) SADP from the BCC region along [012]. The vector g in (**a**) corresponds to the plane normal [$02\bar{1}$]_β_, which is nearly parallel to [100]α. The determined ORs are ($02\bar{1}$)_β_ // (100)_α_ and [$\bar{1}12$]_β_ // [012]α, where “//” denotes nearly parallel.

TEM analysis of the sample held at 550 °C, presented in Figure 6, reveals a distinct orientation relationship (OR) between the BCC matrix and the β-Mn phase. The BF image in Figure 6a shows the coexistence of BCC and β-Mn, with elongated β-Mn precipitates distributed within the matrix in the form of Widmanstätten side-plates. This morphology indicates that the β-Mn phase formed through a diffusion-controlled transformation during isothermal holding, which typically results in precipitates adopting specific orientation relationships with the parent BCC matrix. An SADP obtained from the red-circled region in Figure 6a is shown in Figure 6b. Because this selected area contains both the BCC matrix and the β-Mn precipitate, two distinct diffraction patterns are present: one corresponding to β-Mn and indexed along the β-Mn [$\bar{1}12$] direction and the other corresponding to the α matrix indexed along the BCC [012] direction. To more clearly resolve the diffraction features of each phase, separate SADPs were acquired from regions containing only β-Mn and only α, as shown in Figure 6c and Figure 6d, respectively. The respective zone axes are β-Mn [$\bar{1}12$] (Figure 6c) and BCC [012] (Figure 6d). These results verify the dual-phase structure and the crystallographic correspondence. In Figure 6a, the diffraction vector g, indicated by the arrow, corresponds to the plane normal of [$02\bar{1}$]_β_, which is nearly parallel to [100]α. The indexed reflections in Figure 6b confirm the orientation relationships (ORs) between the two phases as ($02\bar{1}$)_β_ // (100)_α_ and [$\bar{1}12$]_β_ // [012]α, where “//” denotes near-parallel alignment. This ORs indicates a rational lattice correspondence between β-Mn and the BCC matrix, suggesting that β-Mn nucleated heterogeneously along low-interfacial-energy habit planes of the parent phase and maintained partial coherency during growth [2,27,36]. Using the interplanar spacings d_α_ = 0.1450 nm and d_β_ = 0.1409 nm, as calculated for the corresponding planes, the lattice misfit was estimated to be approximately −2.9% for ($02\bar{1}$)_β_ // (100)_α_ the alignment. A misfit of this magnitude falls within the typical range for semi-coherent interfaces (~1–5%), where coherency is locally preserved [33,37].

The orientation relationship (OR) identified in Figure 6 provides insights into the mechanism of β-Mn formation within the BCC matrix. The established OR is ($02\bar{1}$)_β_ // (100)_α_ and [$\bar{1}12$]_β_ // [012]α. This OR indicates a rational lattice correspondence that minimizes both interfacial energy and crystallographic mismatch. Such alignment is characteristic of diffusion-controlled transformations, in which the product phase nucleates on low-interfacial-energy planes of the parent structure [27]. The elongated β-Mn precipitates observed along specific BCC orientations support this interpretation, suggesting that β-Mn formed through Mn and Al interdiffusion while maintaining a partial coherency during growth [2,36]. The interface between the β-Mn and BCC phases exhibits an estimated lattice misfit of approximately −2.9%, suggesting a reasonably good geometric correspondence between the two crystal structures. However, given the complex primitive cubic structure of β-Mn compared with the BCC lattice, full coherency is improbable. The interface is therefore interpreted as semi-coherent.

In summary, while previous investigations emphasized β-Mn formation in austenitic or duplex Fe–Mn–Al alloys, this study extends the understanding to ferritic compositions. The established microstructural evolution behavior, crystallographic orientation relationship, compositional partitioning, and thermal stability range provide a coherent framework for controlling β-Mn precipitation and the overall microstructure of the alloy. These findings provide a framework for tailoring phase stability, morphology, and microstructural evolution in Fe–Mn–Al alloys through controlled thermal processing to enhance alloy performance.

## 4. Conclusions

The present study provides some basic insights into the formation of the β-Mn phase in an Fe–28.6 Mn–10.9 Al alloy subjected to 1100 °C-annealing and subsequent isothermal holding at temperatures ranging from 500 °C to 900 °C. Based on the microstructural analyses, the following conclusions are drawn:

- The alloy exhibits a single BCC phase at temperatures ranging from 850 °C to 1100 °C.
- During isothermal holding, β-Mn precipitates form heterogeneously within the BCC grains and grain boundaries through diffusion-aided elemental partitioning. The compositions of the constituent phases show that the β-Mn phase is enriched in Mn, while the BCC phase is enriched in Fe. Both phases show a weak Al partition.
- The β-Mn phase remains stable in the ferritic phase between approximately 500 °C and 850 °C, with its volume fraction increasing with temperature and reaching a maximum at around 650 °C.
- β-Mn precipitates exhibit a Widmanstätten side-plate morphology, and their sizes increases progressively with the holding temperature.
- The β-Mn and BCC phases maintain an orientation relationship ($02\bar{1}$)_β_ // (100)_α_ and [$\bar{1}12$]_β_ // [012]α, indicating a partially coherent interface between the two structures.
- The disappearance of β-Mn above 850 °C demonstrates its limited thermal stability, indicating that microstructural control in this alloy system requires specific thermal processing.

## Data Availability

The original contributions presented in the study are included in the article. Further inquiries can be directed to the corresponding authors.
